# The quest for biofuels fuels genome sequencing

**DOI:** 10.1111/j.1462-2920.2008.01754.x

**Published:** 2008-10

**Authors:** Michael Y Galperin

**Affiliations:** National Center for Biotechnology Information, National Library of Medicine, National Institutes of HealthBethesda, MD 20894, USA

The list of recently completed microbial genome projects (Table 1) shows further progress in sequencing genomes of poorly studied environmental bacteria. The genome of *Aquifex aeolicus*, sequenced 10 years ago, has been joined by genomes of two more representatives of the phylum *Aquificae*. The genome of *Polaribacter* sp. MED152, a marine member of *Bacteroidetes*, revealed a combination of heterotrophic metabolism with light energy capture by proteorhodopsin. In addition, six genomes from the phylum *Chlorobi* more than doubled the number of sequenced genomes of green sulfur bacteria.

**Table 1:** Recently completed microbial genomes (May–July 2008).

Species name	Taxonomy	GenBank accession	Genome size (bp)	Proteins (total)	Sequencing centre^a^	Reference
New organisms						
*Trichoderma reesei*	Eukaryota, Fungi	AAIL00000000	−34 Mbp	9129	JGI	Martinez *et al.* (2008)
*Kocuria rhizophila*	*Actinobacteria*	AP009152	2 697 540	2357	NITE	Takarada *et al.* (2008)
*Hydrogenobaculum* sp. Y04AAS1	*Aquificae*	CP001130	1 559 514	1629	JGI	Unpublished
*Sulfurihydrogenibium* sp. YO3AOP1	*Aquificae*	CP001080	1 838 442	1721	JGI	Unpublished
*Candidatus* Amoebophilus asiaticus	*Bacteroidetes*	CP001102	1 884 364	1283	JGI	Unpublished
*Polaribacter* sp. MED152	*Bacteroidetes*	NZ_AANA00000000	2 967 150	2646	JCVI	González *et al.* (2008)
*Chlorobaculum parvum*	*Chlorobi*	CP001099	2 289 249	2043	JGI	Unpublished
*Chlorobium limicola*	*Chlorobi*	CP001097	2 763 181	2434	JGI	Unpublished
*Chloroherpeton thalassium*	*Chlorobi*	CP001100	3 293 456	2710	JGI	Unpublished
*Pelodictyon phaeoclathratiforme*	*Chlorobi*	CP001110	3 018 238	2707	JGI	Unpublished
*Prosthecochloris aestuarii*	*Chlorobi*	CP001108	2 512 923	2327	JGI	Unpublished
		CP001109	66 772			
*Natranaerobius thermophilus*	*Firmicutes*	CP001034	3165 557	2906	JGI	Unpublished
		CP001035	17 207			
		CP001036	8 689			
*Methylobacterium populi*	*α-Proteobacteria*	CP001029	5 800 441	5365	JGI	Unpublished
		CP001030	25 164			
		CP001031	23 392			
*Oligotropha carboxidovorans*	*α-Proteobacteria*	ABKN00000000	3 745 772	3754	Mississippi State U.	Paul *et al.* (2008)
*Wolbachia pipientis*	*α-Proteobacteria*	AM999887	1 482 455	1275	Sanger Institute	Klasson *et al*. (2008)
*Ralstonia pickettii*	*β-Proteobacteria*	CP001068	3 942 557	4952	JGI	Unpublished
		CP001069	1 302 238			
		CP001070	80 934			
*Cellvibrio japonicus*	*γ-Proteobacteria*	CP000934	4 576 573	3754	JCVI	DeBoy *et al.* (2008)
*Erwinia tasmaniensis*	*γ-Proteobacteria*	CU468128	4.07 (total)	3622	MPIMG	Kube *et al.* (2008)
		CU468130–CU468135				
*Proteus mirabilis*	*γ-Proteobacteria*	AM942759	4 063 606	3685	Sanger Institute	Pearson *et al.* (2008)
		AM942760	36 289			
*Geobacter lovleyi*	*δ-Proteobacteria*	CP001089	3 917 761	3476	JGI	Unpublished
		CP001090	77 113			
*Candidatus* Phytoplasma mali	*Tenericutes*	CU469464	601 943	479	MPIMG	Kube *et al*. (2008)
*Mycoplasma arthritidis*	*Tenericutes*	CP001047	820 453	631	JCVI	Dybvig *et al*. (2008)
New strains						
*Bifidobacterium longum* DJO10A	*Actinobacteria*	CP000605	2 375 792	2003	JGI	Lee *et al*. (2008)
		AF538868	10 073			
		AF538869	3 661			
*Chlorobium phaeobacteroides*BS1	*Chlorobi*	CP001101	2 736 403	2469	JGI	Unpublished
*Lactobacillus casei*BL23	*Firmicutes*	FM177140	3 079 196	3044	INRA	Unpublished
*Streptococcus pneumoniae*G54	*Firmicutes*	CP001015	2 078 953	2115	JCVI	Dopazo *et al.* (2001)
*Rhizobium etli* CIAT 652	*α-Proteobacteria*	CP001074–CP001077	6.44 (total)	6056	UNAM	Unpublished
*Rhodopseudomonas palustris* TIE-1	*α-Proteobacteria*	CP001096	5 744 041	5246	JGI	Unpublished
*Burkholderia cenocepacia*J2315	*β-Proteobacteria*	AM747720–AM747723	8.05 (total)		Sanger Institute	Unpublished
*Burkholderia multivorans* ATCC 17616	*β-Proteobacteria*	AP009385–AP009388	6.99 (total)	6112	Tohoku U.	Unpublished
*Neisseria gonorrhoeae*NCCP11945	*β-Proteobacteria*	CP001050	2 232 025	2674	Korea NIH	Chung *et al*. (2008)
		CP001051	4 153			
*Actinobacillus pleuropneumoniae* serovar 7 str. AP76	*γ-Proteobacteria*	CP001091–CP001094	2.34 (total)	2142	Bielefeld U.	Unpublished
*Salmonella enterica* subsp. *enterica* serovar Heidelberg str. SL476	*γ-Proteobacteria*	CP001120	4 888 768	4779	JCVI	Unpublished
		CP001118	91 374			
		CP001119	3 373			
*Salmonella enterica* subsp. *enterica* serovar Newport str. SL254	*γ-Proteobacteria*	CP001113	4 827 641	4805	JCVI	Unpublished
		CP000604	176 473			
		CP001112	3 605			
*Salmonella enterica* subsp. *enterica* serovar	*γ-Proteobacteria*	CP001127	4 709 075	4627	JCVI	Unpublished
Schwarzengrund str. CVM19633		CP001125	110 227			
		CP001126	4 585			
*Stenotrophomonas maltophilia*R551-3	*γ-Proteobacteria*	CP001111	4 573 969	4039	JGI	Unpublished
*Treponema pallidum* subsp. *pallidum*SS14	*Spirochaetes*	CP000805	1 139 457	1028	Baylor	Matejkova *et al*. (2008)

Sequencing centre names are abbreviated as follows: Baylor, Human Genome Sequencing Center, Baylor College of Medicine, Houston, Texas, USA; Bielefeld U., Centrum für Biotechnologie, Universität Bielefeld, Bielefeld, Germany; INRA, Institut National de la Recherche Agronomique, Domaine de Vilvert, Jouy en Josas, France; JCVI, J. Craig Venter Institute, Rockville, Maryland, USA; JGI, US Department of Energy Joint Genome Institute, Walnut Creek, California, USA; Korea NIH, Center for Infectious Disease and Research, Korea National Institute of Health, Seoul, Korea; Mississippi State U., Mississippi State University, Mississippi State, Mississippi, USA; MPIMG, Max-Planck-Institute for Molecular Genetics, Berlin, Germany; NITE, Genome Analysis Center, Department of Biotechnology, National Institute of Technology and Evaluation, Shibuya-ku, Tokyo, Japan; Sanger Institute, The Wellcome Trust Sanger Institute, Wellcome Trust Genome Campus, Hinxton, Cambridgeshire, UK; Tohoku U., Department of Environmental Life Sciences, Graduate School of Life Sciences, Sendai, Miyagi, Japan; UNAM, Centro de Ciencias Genomicas, Universidad Nacional Autonoma de Mexico, Cuernavaca, Mexico.

In eukaryotic genomics, important news was the release by the JGI scientists of a draft genome of the soft-rot ascomycete fungus *Trichoderma reesei*, also known as *Hypocrea jecorina* (Martinez *et al*., 2008). *Trichoderma reesei* is filamentous fungus that is widely used in biotechnology as a producer of various cellulases and hemicellulases for the hydrolysis of plant cell walls. This organism has attracted renewed interest owing to its potential use in the conversion of lignocelluloses to biofuel. The GenBank version of the draft genome of *T. reesei* consists of 2236 contigs, assembled into 170 scaffolds and containing ∼34 Mbp of DNA, representing ∼99% of the whole genome. The current assembly did not assign the scaffolds to any of the seven chromosomes of *T. reesei*, but allowed identification of 9129 predicted protein-coding genes (Martinez *et al*., 2008). Comparison of *T. reesei* with *Fusarium graminearum* (*Gibberella zeae*) and *Neurospora crassa* revealed a certain degree of synteny between these three genomes. A surprising finding was the relatively low number of glycoside hydrolases (cellulases, hemicellulases and pectinases) encoded by *T. reesei* genome. The authors suggest that successful utilization by *T. reesei* of its limited set of cellulolytic enzymes to efficiently degrade plant cell walls could be due to (i) clustering of the respective genes that ensures co-expression of the right combination of hydrolytic enzymes, and (ii) secretion of secondary metabolites (Martinez *et al*., 2008).

Although phylogenetically unrelated to *T. reesei*, the γ-proteobacterium *Cellvibrio japonicus* also encodes an efficient machinery for degrading plant cell walls that includes 130 predicted glycoside hydrolases (DeBoy *et al*., 2008).

The current list includes two actinobacterial genomes, representing the soil bacterium *Kocuria rhizophila* (Takarada *et al*., 2008) and a new strain of the human gut symbiont *Bifidobacterium longum* (Lee and O'Sullivan, 2006; Lee *et al*., 2008)*.* The genus *Kocuria* belongs to the family *Micrococcineae* and was separated from *Micrococcus* just a few years ago (Stackebrandt *et al*., 1995). Accordingly, *K. rhizophila* ATCC 9341, parental strain of the sequenced *K. rhizophila* DC2201, was until recently classified as *Micrococcus luteus* and used as a standard quality control strain in a number of applications, including testing of antimicrobial compounds (Tang and Gillevet, 2003). The genus name was assigned to honour Miroslav Kocur, Slovakian microbiologist who dedicated many years to studying *M. luteus* (Rosypal and Kocur, 1963; Kocur, 1986). *Kocuria rhizophila* is an environmental actinomycete that is often associated with plant roots. Despite its small (for a soil actinomycete) 2.7 Mbp genome, *K. rhizophila* appears to encode the full set of key metabolic enzymes. However, it encodes fewer proteins participating in secondary metabolism, including single genes for a non-ribosomal peptide synthetase and a polyketide synthase. The relatively high tolerance of *K. rhizophila* to various organic compounds correlates with the presence of a large number of genes encoding various membrane transporters, including drug efflux pumps (Takarada *et al*., 2008).

The two newly sequenced genomes of *Aquificae* represent two major families in this phylum. *Hydrogenobaculum* sp. YO4AAS1 belongs to the family *Aquificaceae*, which also includes *A. aeolicus*, the best-characterized member of the phylum, whereas *Sulfurihydrogenibium* sp. YO3AOP1 belongs to the family *Hydrogenothermaceae*. Both are thermophilic chemolitoautotrophs, isolated from hot springs at Yellowstone National Park at 60–75°C and capable of growing in microaerophilic conditions by using reduced sulfur compounds and/or hydrogen as electron acceptors and CO_2_ as the source of carbon (Stöhr *et al*., 2001; Reysenbach *et al*., 2005). However, the former is an acidophile, growing at or below pH 3.0, and the latter grows at neutral pH values. The genome size of *Hydrogenobaculum* sp. YO4AAS1 is very close to that of *A. aeolicus*, whereas *Sulfurihydrogenibium* sp. YO3AOP1 features a 300 kb larger genome and almost a hundred of extra proteins. Availability of these new genomes should provide a much-needed insight into the physiology of *Aquificae*, one of the earliest-branching bacterial lineages.

Of the two members of the highly diverse phylum *Bacteroidetes* in the current list, the first one, *Candidatus* Amoebophilus asiaticus, is an obligate intracellular symbiont of the amoebae *Acanthamoeba* sp. (Horn *et al*., 2001). However, it has a much larger genome and encodes far more proteins than *Candidatus* Sulcia muelleri, another member of the *Bacteroidetes* that is an endosymbiont of sharpshooters (McCutcheon and Moran, 2007). In addition, JGI scientists plan to sequence the genome of *Candidatus* Cardinium hertigii, a symbiont of *Encarsia* wasps. Comparison of *Ca.* A. asiaticus with *Ca*. S. muelleri and *Ca.* C. hertigii on one hand and to free-living *Bacteroidetes* on the other should provide further clues to the mechanisms of bacterial adaptation to the endosymbiotic lifestyle.

The second *Bacteroidetes* member, *Polaribacter* sp. MED152, is a marine bacterium that was isolated from the surface water of north-western Mediterranean Sea off the Catalan coast (González *et al*., 2008). In the original GenBank submission, it was listed as a strain of *Polaribacter dokdonensis* (Yoon *et al*., 2006), with which it shares 99.6% similar 16S rRNA sequence. However, because of certain phenotypic differences between the two, the authors have chosen to refer to the sequenced organism simply as ‘strain MED152’. Together with the previously described *Gramella forsetii* (Bauer *et al*., 2006), *Polaribacter* sp. MED152 represents the marine *Bacteroidetes* that in certain conditions may comprise up to 20% of the bacterioplankton. Physiology of these bacteria is still poorly understood, and the authors use the genome of MED152 to offer a very attractive scheme of a ‘dual lifestyle’ for this organism. Based on the abundance of protease and glycosidase genes, they propose that the normal *modus operandi* for MED152 includes gliding motility in search for suitable polymers and their subsequent degradation for carbon, nutrients and energy (González *et al*., 2008). However, once suitable polymeric substrates have been exhausted, MED152 must sustain itself in a nutrient-poor environment. In contrast to *G. forsetii*, MED152 encodes proteorhodopsin, an H^+^-translocating light-dependent ion pump that can use light energy to charge the membrane, generating the proton-motive force. In fact, exposure to light does not stimulate growth of MED152 but appears to stimulate bicarbonate uptake and, conceivably, assimilation of carbon dioxide (González *et al*., 2008). Accordingly, MED152 encodes a variety of (predicted) light sensors that have not been seen in other members of *Bacteroidetes*. As noted in the accompanying insightful comment (Kirchman, 2008), the ability of marine bacteria to absorb light and use it to supplement their energy needs has important consequences for the understanding of the global carbon cycle.

In the past 2 months, JGI scientists released six complete genomes of *Chlorobi* (green sulfur bacteria), five of which, *Chlorobaculum parvum*, *Chlorobium limicola*, *Chloroherpeton thalassium*, *Pelodictyon phaeoclathratiforme* and *Prosthecochloris aestuarii*, represent new species and one, *Chlorobium phaeobacteroides* represents a new strain of the species that had its first sequenced genome 2 years earlier (Table 1). Like other green sulfur bacteria, all these strains are anoxygenic phototrophs that live in strictly anaerobic sulfide-rich environments. They gain energy from photosynthesis, which relies on type I reaction centres and uses sulfide, sulfur and/or thiosulfate as electron acceptors, and fix carbon through the reverse TCA cycle (Overmann and Garcia-Pichel, 2000; Frigaard and Bryant, 2004). The species differ in their ecological niches and the relative amounts of carotene pigments and bacteriochlorophylls *a*, *c*, *d* and *e*. Green sulfur bacteria play a key role in carbon, nitrogen and sulfur turnover in anoxic freshwater aquatic environments and are a potential source of biomass for biofuels. In addition, *Prosthecochloris aestuarii*, which forms multilayered biofilms, has been implicated in microbial infection of coral reefs. Comparative analysis of these genomes should clarify many unanswered questions in physiology of these interesting and important organisms.

*Natranaerobius thermophilus* strain JW/NM-WN-LF is an anaerobic, halophilic alkalithermophile isolated from sediments of a solar-heated, alkaline, hypersaline soda lake at Wadi An Natrun, Egypt (Mesbah *et al*., 2007). Its optimum growth conditions are 53°C, pH 9.5 and between 3.3 and 3.9 M Na^+^. It cannot grow at pH lower than 8.3 (or higher than 10.8). This organism belongs to a separate lineage in the class *Clostridia* and is currently assigned to the separate order *Natranaerobiales* and family *Natranaerobiaceae*. A detailed analysis of its genome sequence should clarify the adaptations of *N. thermophiles* to its unique ecological niche but it is already obvious that they include a Na^+^-dependent F_1_F_O_-type ATP synthase, very similar to the ones in the recently sequenced genomes of *Alkaliphilus metalliredigens* and *Alkaliphilus oremlandii*.

Other organisms with newly sequenced genomes include the chemolithoautotrophic α-proteobacterium *Oligotropha carboxidovorans* (Paul *et al*., 2008)*,* copper-resistant β-proteobacterium *Ralstonia pickettii* 12J, plant epiphyte *Erwinia tasmaniensis* (a non-pathogenic relative of widespread plant pathogens (Kube *et al*., 2008b), endophytes of the poplar tree *Methylobacterium populi* (Van Aken *et al*., 2004) and *Stenotrophomonas maltophilia* R551-3, tetrachloroethene-dechlorinating δ-proteobacterium *Geobacter lovleyi* (Sung *et al*., 2006; Strycharz *et al*., 2008), new strains of *Rhizobium etli*, *Treponema pallidum* and many others (Table 1).

The current list also includes genomes of two mollicutes, *Candidatus* Phytoplasma mali and *Mycoplasma arthritidis*. The first one is a phytopathogen infecting apple, cherry, apricot and plum trees. It was isolated in Heidelberg, Germany, from an apple tree displaying symptoms of apple proliferative disease and is the first mycoplasma to have a linear chromosome (Kube *et al*., 2008a). The second one causes arthritis in rats and mice and is remarkable for carrying a lysogenic bacteriophage (Dybvig *et al*., 2008).

However, the greatest surprise in the mycoplasma studies came not from genome sequencing labs but from taxonomists. Although mycoplasmas have long been listed in the Division *Tenericutes* (International Committee on Systematic Bacteriology-Subcommittee on the Taxonomy of Mollicutes, 1995), this clade was usually considered together with *Rickettsia* and *Chlamydia* and not treated as an actual taxonomic unit. Instead, *Mollicutes* were considered a class in the phylum *Firmicutes*, which was consistent with the available phylogenetic analyses (Falah and Gupta, 1997; Ciccarelli *et al*., 2006). However, in the recent edition of *Bergey's Manual of Systematic Bacteriology*, class Mollicutes was excluded from the phylum *Firmicutes* and moved to the new phylum *Tenericutes* (Ludwig *et al*., 2008). While there might have been valid reasons for doing that (for example, many mycoplasma use a non-standard genetic code with UGA codon coding for tryptophan instead of terminating translation), the cited reason for that move was comparative analysis of mycoplasmal sequences by Ludwig and Schleifer (2005), published in a book to which many researchers had no access. Given that the goal of *Bergey's Manual* is introduction of ‘phylogenetic framework’ (Ludwig *et al*., 2008), it seems unfortunate that such important changes are being made without a public discussion or at least a publication in a peer-reviewed journal. After all, massive investments in microbial genome sequencing worldwide have moved bacterial taxonomy from a purely academic sphere into the realm of the biotechnological marketplace, and relatively minor changes in classification could have serious effect on the priorities in future genome sequencing projects.
